# Head and neck cancer of unknown primary: unveiling primary tumor sites through machine learning on DNA methylation profiles

**DOI:** 10.1186/s13148-024-01657-3

**Published:** 2024-03-25

**Authors:** Leonhard Stark, Atsuko Kasajima, Fabian Stögbauer, Benedikt Schmidl, Jakob Rinecker, Katharina Holzmann, Sarah Färber, Nicole Pfarr, Katja Steiger, Barbara Wollenberg, Jürgen Ruland, Christof Winter, Markus Wirth

**Affiliations:** 1 Department of Otolaryngology, Head and Neck Surgery, School of Medicine and Health, Technical University of Munich, Munich, Germany; 2 Institute of Clinical Chemistry and Pathobiochemistry, School of Medicine and Health, Technical University of Munich, Munich, Germany; 3 Institute of Pathology, School of Medicine and Health, Technical University of Munich, Munich, Germany; 4 Center for Translational Cancer Research, TranslaTUM, Technical University of Munich, Munich, Germany; 5 Partner Site Munich and German Cancer Research Center (DKFZ), German Cancer Consortium (DKTK), Heidelberg, Germany

**Keywords:** CUP, HNSCC, DNA methylation, Classifier

## Abstract

**Background:**

The unknown tissue of origin in head and neck cancer of unknown primary (hnCUP) leads to invasive diagnostic procedures and unspecific and potentially inefficient treatment options for patients. The most common histologic subtype, squamous cell carcinoma, can stem from various tumor primary sites, including the oral cavity, oropharynx, larynx, head and neck skin, lungs, and esophagus. DNA methylation profiles are highly tissue-specific and have been successfully used to classify tissue origin. We therefore developed a support vector machine (SVM) classifier trained with publicly available DNA methylation profiles of commonly cervically metastasizing squamous cell carcinomas (*n* = 1103) in order to identify the primary tissue of origin of our own cohort of squamous cell hnCUP patient’s samples (*n* = 28). Methylation analysis was performed with Infinium MethylationEPIC v1.0 BeadChip by Illumina.

**Results:**

The SVM algorithm achieved the highest overall accuracy of tested classifiers, with 87%. Squamous cell hnCUP samples on DNA methylation level resembled squamous cell carcinomas commonly metastasizing into cervical lymph nodes. The most frequently predicted cancer localization was the oral cavity in 11 cases (39%), followed by the oropharynx and larynx (both 7, 25%), skin (2, 7%), and esophagus (1, 4%). These frequencies concord with the expected distribution of lymph node metastases in epidemiological studies.

**Conclusions:**

On DNA methylation level, hnCUP is comparable to primary tumor tissue cancer types that commonly metastasize to cervical lymph nodes. Our SVM-based classifier can accurately predict these cancers’ tissues of origin and could significantly reduce the invasiveness of hnCUP diagnostics and enable a more precise therapy after clinical validation.

**Supplementary Information:**

The online version contains supplementary material available at 10.1186/s13148-024-01657-3.

## Introduction

### Head and neck cancer of unknown primary

Head and neck cancer is a group of malignant tumors originating from the oral cavity, pharynx, larynx, salivary glands, sinuses, nose, or head and neck skin. Head and neck squamous cell carcinoma (HNSCC) is the most common subtype and accounts for approximately 90% of head and neck cancer cases [1]. Head and neck cancer of unknown primary (hnCUP) is defined as lymph node metastasis in the head and neck region without a corresponding primary tumor [2]. In about 50% of initial hnCUP cases, the primary tumor can be found through a multi-step diagnostic workup [3]. Unresolved hnCUP cases amount to 1.5–3% of head and neck cancer patients and have a 5-year survival rate of 30–40% [2]. The most common histologic subtype (around two-thirds) is the squamous cell carcinoma (SCC) [4–7].

Although there is, by definition, no way to validate the primary sites of hnCUP, it is conceivable that they show a distribution similar to that of typically cervically metastasizing cancers. CUP can also possibly be an entirely unique entity [8]. However, the most likely explanation for most CUP cases suggests the existence of a distinct but small primary tumor, which may be detected by a thorough diagnostic workup [8]. This hypothesis is partly supported by the metachronous identification of the primary tumor in 57% of patients who did not receive adjuvant radiotherapy to potential primary sites in one study [2]. In these cases, a small primary tumor grows without treatment until it is detected in clinical examination or imaging.

To evaluate CUP as a possible separate entity, limited investigations of the mutational landscape have been performed [9, 10]. Gottschlich et al., for example, showed a significant reduction in TP53 mutations in 23 hnCUP samples compared to the expected frequency in SCC of the head and neck [9]. However, other studies could not find any significant differences in mutation and protein expression analyses between CUP cases and cases with known primary tumor [10]. CUP itself has been shown to have a very heterogeneous mutational landscape [11], like metastatic and late-stage tumors.

Considering the ambivalent data from rather small study cohorts supporting CUP being a unique cancer type [12], it is most commonly assumed to be a metastatic disease stemming from a broad variety of different cancer entities [10, 13]. This hypothesis is further supported by successful approaches to demarcate the primary tumor sites based on DNA methylation analysis of CUP, as will be discussed below [14, 15].

### DNA methylation profiles for the classification of tumor tissue

DNA methylation has been successfully used for the classification of tumor tissue samples [16–19]. The tissue specificity of DNA methylation also enabled the identification of the origin of tissue. Relevant examples are:

Moran et al. implemented a methylation-based classifier that correctly predicts the different unknown primary sites of CUP tumors. Freely available methylation data of various tumor entities provided by The Cancer Genome Atlas (TCGA) were used to train the prediction model. This classifier could distinguish between 38 cancer types accurately [11].

The distinction between pulmonary metastases and head and neck cancer (HNSCC) has been demonstrated by Jurmeister et al. using DNA methylation analyses [20]. In another work, Leitheiser et al. were able to predict the tissue origin of lymph node metastasis samples of HNSCC [14].

### Origin of cervical lymph node metastasis

**Fig. 1:**
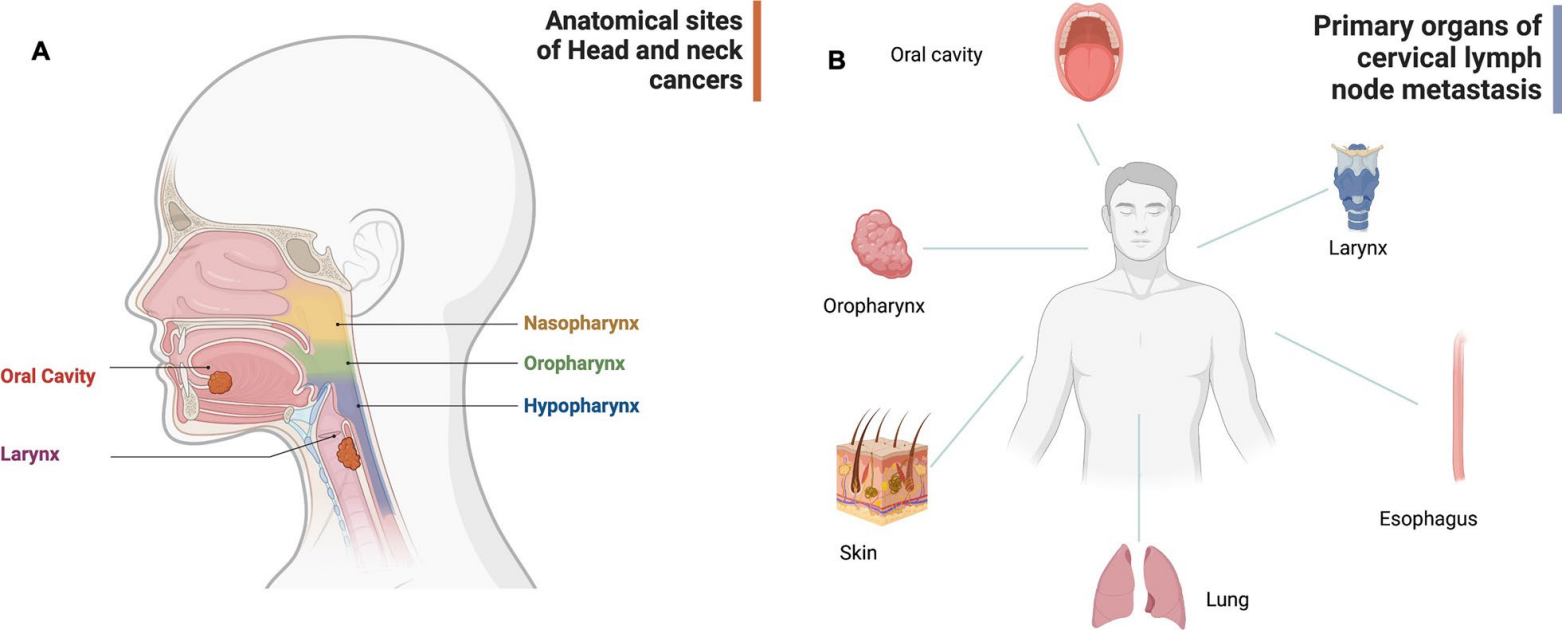
Anatomy of head and neck cancer and cervical lymph node metastasis. **A** Anatomical sites of head and neck cancers. **B** Primary organs of cervical lymph node metastasis

Despite promising molecular techniques, the diagnosis of an SCC lymph node metastasis without a corresponding primary still poses a diagnostic dilemma as the entity cannot be distinguished with routine pathological examination [20]. In most cases, HNSCC are responsible for cervical lymph node metastases. However, esophageal SCC (ESCC), lung SCC (LUSCC), and cutaneous squamous cell carcinoma (CSCC) do contribute to cervical lymph node metastases as further possible tumor entities (Fig. 1). Epidemiological data of these entities allow us to estimate how likely they are to metastasize cervically [21–26].

This study aimed to analyze whether methylation profiles obtained from lymph node tissue of hnCUP patients resemble or differ from those of tumors that can metastasize into head and neck lymph nodes, not only focusing on HNSCC but also on head and neck skin, lung, and esophagus SCC. Another objective was to explore potential variations in the frequency distribution between hnCUP and lymph node metastases with confirmed primary tumors.

## Materials and methods

### Study cohort

We retrospectively selected 28 patients (median age 64; 4 females, 24 males) with hnCUP from a preexisting cohort at the Department of Otorhinolaryngology, Head and Neck Surgery, Technical University of Munich, Germany, who underwent treatment between 2002 and 2013. The primary tumor site of the metastases was not found despite an exhaustive diagnostic workup consisting of a thorough clinical examination, panendoscopy of the oral cavity, pharynx, and larynx, as well as multimodal imaging comprising PET-CT, CT, and MRI scans. All samples were seen by a pathologist and confirmed as SCC histologically. Table 1 provides an overview of the study cohort’s demographic and clinical characteristics, while (see Additional file: 2) offer additional clinical features.

**Table 1:** Clinical characteristics of hnCUP patients (study cohort)

Characteristic	Total *n* = 28			
Age, median (IQR)	64 (56–71)			
Sex	Male = 24	Female = 4		
Nicotine consumption	Never = 6	Smoker = 16	Ex-smoker = 3	Unknown = 3
p16 positivity > 70%	Pos = 8	Neg = 20		
*N* status	N1 = 2	N2 = 24	N3 = 2	
*M* status	M0 = 23	M1 = 2	Mx = 3	
Grading	G1 = 1	G2 = 10	G3 = 17	

For each patient, lymph node tissue was obtained by lymph node extirpation. Tissue was then formalin-fixed and paraffin-embedded (FFPE). Patients received no therapy prior to tissue sampling. The study has received approval from the local ethics committee (Technical University of Munich, 285/20 S-KH).

### Reference cohort from publicly available data

For classifier development, we used publicly available as well as our data obtained through DNA methylation profiling of primary tumors and lymph node metastases (Table 2). Sources included the TCGA by the National Institutes of Health (NIH), the German Cancer Research Center (Deutsches Krebsforschungszentrum, DKFZ) in Heidelberg, and the Charité Medical School in Berlin.

**Table 2:** Data sets used for visualization and classifier development

Source	Samples	References	Chip type (k)	Tumor entity	Tissue type	Tissue origin
TCGA-HNSC	518	NIH [27]	450	HNSCC	Fresh frozen	Primary tumor
TCGA-ESCA	95	NIH [27]	450	ESCC	Fresh frozen	Primary tumor
TCGA-LUSC	405	NIH [27]	450	LUSCC	Fresh frozen	Primary tumor
TCGA-DLBC	48	NIH [27]	450	DLBC	Fresh frozen	Primary tumor
DKFZ Heidelberg	18	Paredes et al. [28]	EPIC (850)	CSCC	FFPE	Primary tumor
DKFZ Heidelberg	19	Koelsche et al. [29]	EPIC (850)	CSCC	FFPE	Primary tumor
Charité Berlin	49	Leitheiser et al. [14]	EPIC (850)	HNSCC	FFPE (lymph node)	Lymph node metastasis
Klinikum rechts der Isar Munich	28	This study	EPIC (850)	hnCUP	FFPE (lymph node)	Lymph node metastasis

The primary tumor sites of cancers typically causing cervical lymph node metastases were considered. HNSCC, ESCC, CSCC, and LUSCC histologically match SCCs, whereas the diffuse large B-cell lymphoma (DCBL) samples were chosen for comparison. While being a potential primary site for hnCUP, the hypopharynx was excluded as a primary site since only ten samples were available from TCGA. Primary tumor tissue samples were used in most of the data sets despite the study cohort containing lymph node tissue samples. We made this decision because there is significantly more publicly available data from primary tumor samples than lymph node samples.

The data sets from our reference cohort are shown in Table 3.

**Table 3:** Distribution of primary tumor sites in the reference cohort

Primary tumor site	Number of samples
Esophagus	95
Larynx	117
Lung	405
Lymphoma	48
Oral cavity	320
Oropharynx	81
Skin	37
Overall	1103

For further analyses, the available data sets were randomly distributed into a training cohort and a test cohort. These consist of two-thirds and one-third of the patients, respectively, and share the same proportions of primary tumor sites.

### Sample processing of study cohort

From FFPE tissue samples, areas containing at least 50% tumor cells were identified and marked by a pathologist. After macrodissecting these marked areas, DNA was extracted using the Maxwell RSC Blood DNA Kit with the Maxwell RSC 48 instrument (Promega). For DNA quality control, fragmentation and integrity were evaluated using the 4200 TapeStation system (Agilent), and DNA levels were quantified with the Qubit 4 Fluorometer (Thermo Fischer Scientific).

Subsequently, DNA methylation profiles were generated in collaboration with the DKFZ in Heidelberg. The Microarray Unit of the Genomics and Proteomics Core Facility at the DKFZ has established a DNA methylation analysis workflow using FFPE tissue which was utilized in previous projects with high quality [16]. Using bisulfite treatment, the non-methylated cytosine bases of the DNA are converted to uracil and can thus be distinguished from methylated cytosine bases, which are not converted. The Human MethylationEPIC v1.0 BeadChip by Illumina was used to generate the profiles. A separate slot on the BeadChips, holding up to 8 samples, was used for each.

### Methylation data processing

The Illumina MethylationEPIC BeadChip, which covers about 850,000 CpG sites, was used for samples from hnCUP, CSCC, and Leitheiser et al.’s HNSCC cohort. All other samples were processed using the Illumina 450 k chip.

DNA methylation analysis was performed using the programming language R and the *minfi* package [30] to combine data sets of Illumina MethylationEPIC (850 k) and Illumina 450 k methylation arrays. We used the IDAT files generated by the Illumina microarrays as input data. IDAT (intensity data) files are generated by microarray-based gene expression profiling technologies and contain the raw fluorescence intensity data for each probe on the microarray, which can be used to quantify gene expression levels. As suggested by Fortin et al. [31], we performed the single-sample Noob (ssNoob) method to preprocess samples for optimal cross-array normalization. The combined arrays contain beta values of 452,453 CpG sites for all 1131 samples. Normalization was performed for the training, test, and study cohorts, respectively. Additional file: 6 contains the R script used for preprocessing.

For further analyses, we selected the CpG sites with the highest standard deviation of their beta values across all samples. We aimed to choose as few sites as possible while still maintaining a high prediction accuracy of the machine learning classifiers. After exploring CpG amounts ranging from 10 to 15,000, we decided on a final set of 3,000 CpG sites, found in the tables (Additional file: 3). Higher amounts of CpG sites used as input did not result in better performance of the methods used.

### UMAP plots and clustering

To visualize the DNA methylation profiles of both the study cohort and the publicly available data sets, uniform manifold approximation and projection (UMAP) was employed. Clusters of SCC DNA methylation profiles obtained from samples can be visualized by reducing the high-dimensional methylation array data to two dimensions. All samples were annotated with clinical data from the corresponding data set (Table 2). The *umap* package was used to compute the reference cohort’s data to be plotted. The R package *pheatmap* was used to generate heatmaps of the methylation profiles. The R script used to generate the plots in the manuscript can be found in Additional file: 7.

Various dimensionality reduction techniques were considered, including principal component analysis (PCA), *t*-distributed stochastic neighbor embedding (*t*-SNE), and UMAP. After careful evaluation, we adopted UMAP, as it consistently produced plots with distinct and quickly computable clusters.

### Classifier development

We evaluated support vector machines (SVMs) and random forest classifiers to determine which algorithm would be better suited for class prediction. The random forest classifier used node sizes of 1, 2, 3, 10, and 50. To measure the performance of the resulting classifiers, we used overall balanced accuracy defined as (sensitivity + specificity) / 2. For binary classification methods such as SVMs, we combined multiple one-vs-rest classifiers into a metaclassifier for multiple class prediction using the *R* package *caret* (see Additional file: 8 for the R script). The training was performed on the training cohort (two-thirds split of the reference cohort). The classification models were then tested with the test cohort (one-third split of the reference cohort).

## Results

### The origin distribution of cervical lymph node metastases can be calculated using tumor incidences from the literature

Since the aim of this study was to analyze whether methylation profiles obtained from lymph node tissue of the neck region can identify the tissue of origin for hnCUP patients, we started by estimating the relative contributions to lymph node metastases of different primary tumor sites. Metastatic disease of the head and neck region that histologically matches SCC has a variety of possible underlying primary tumor sites. HNSCC is the most common cause, but other causes include ESCC, LUSCC, and CSCC. To get a better understanding of the epidemiology of cervical lymph node metastases, we queried PubMed using each tumor entity combined with the keywords “lymph node,” “metastasis,” and “epidemiology.” We used the overall worldwide incidences and rates of cervical metastases of selected cancer entities to estimate the composition of potential origins (Table 4). For non-melanoma skin cancer, only cases concerning the head and neck area (55%) were considered [32]. While, as expected, head and neck tumors account for the majority of metastatic lymph node cases, esophageal cancer (ESCC) accounts for around one-fifth of the cases.

**Table 4:** Incidences of different tumor entities that metastasize to lymph nodes in the head and neck region

Tumor entity	Incidence (worldwide 2020)	SCC proportion	Incidence SCC	Proportion of cervical metastasis	Incidence of cervical metastasis	Proportion of all cervical metastases	References
Lung cancer	2,206,771	27.5%	606,862	1.5%	9,103	2%	[21]
ESCC	604,000	100%	604,000	18.1%	109,324	22%	[22, 23]
HNSCC—oral cavity	377,713	100%	377,713	60%	226,628	45%	[24]
HNSCC—larynx	184,615	100%	184,615	50%	92,308	18%	[25]
HNSCC—oropharynx	98,412	100%	98,412	60%	59,047	12%	[24]
Non-melanoma skin cancer	1,198,073	13.8%	1,198,073	4%	6,589	1%	[26]
Total					502,999	100%	

Incidence numbers were taken from the Global Cancer Observatory [19]. The proportion of cervical metastasis was approximated based on relevant literature, as noted in the reference column

### DNA methylation-based clustering of tumor samples reveals tissue of origin

**Fig. 2:**
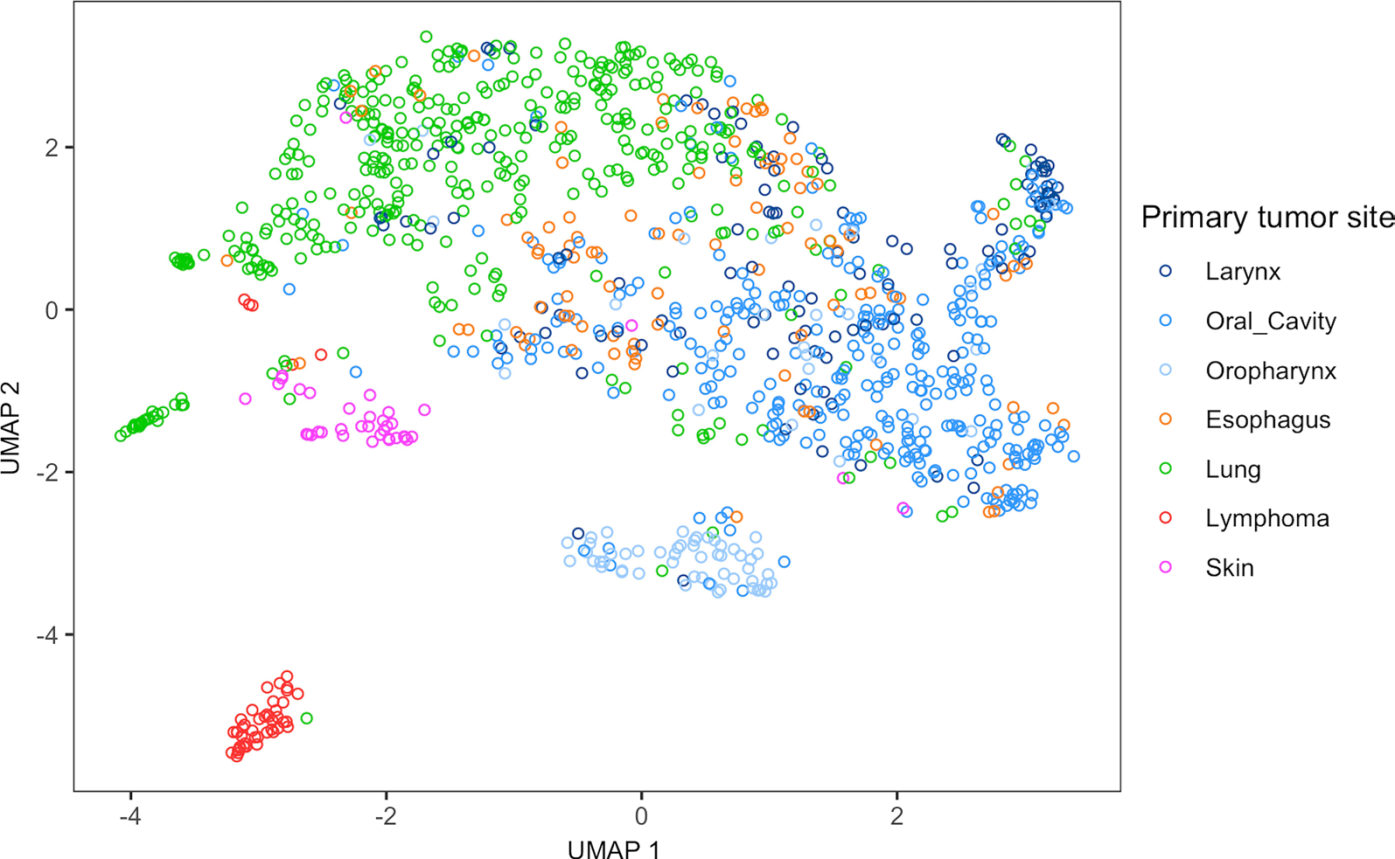
UMAP clustering of DNA methylation profiles of all samples of the reference cohort (*n* = 1103). All of these entities typically metastasize to cervical lymph nodes

**Fig. 3:**
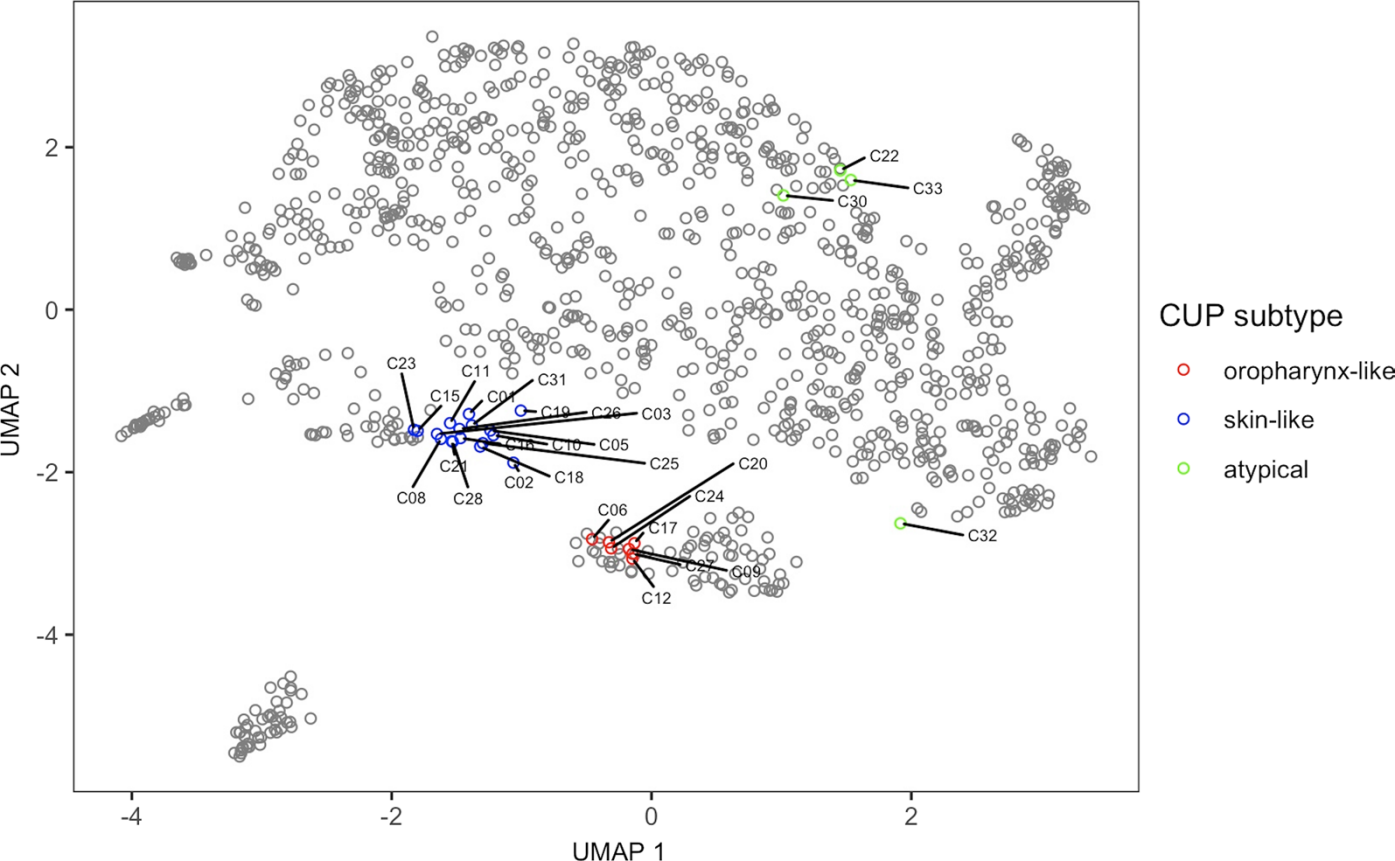
UMAP clustering of the reference and study cohort. In addition to the samples in Fig. 2, 28 hnCUP DNA methylation profiles from the study cohort are included. hnCUP samples are marked with their sample IDs. The hnCUP samples have been assigned to the oropharynx cluster (red), skin cluster (blue), or denoted as atypical samples (green) based on their relative spatial proximity in the UMAP clustering on visual inspection and further confirmed by hierarchical clustering (Fig. 4)

For clustering, DNA methylation profiles (*n* = 1103) of cervically metastasizing SCC were obtained from publicly available sources (reference cohort, see Table 2). We employed uniform manifold approximation and projection (UMAP) analysis of all reference cohort samples, resulting in several distinct clusters forming (Fig. 2). Plots of PCA and *t*-SNE analyses are deposited in Additional file: 1 for comparison. As a control, we added lymphoma samples, which, being from lymphoid origin, formed a separate distinct cluster, indicating a clear difference to the squamous cell carcinomas on a level of DNA methylation. Notably, most oropharynx samples form one cluster. Another distinguishable group consists of the CSCC samples (Fig. 2, depicted in red). Next, we added squamous cell hnCUP samples (*n* = 28, study cohort) and clustered these in comparison in Fig. 3. Some of our hnCUP samples also fall into these two groups. Samples from the remaining primary sites form a large cluster and can graphically be distinguished to some extent. Plots of just the training set showing the samples’ true entity of origin and the test set with predicted primary sites are shown in Additional file: 1. These indicate a similar distribution.

Based on DNA methylation, CUP forms no separate cluster but rather co-clusters with cancers of the head and neck, skin, lung, and esophagus. The hnCUP samples of the study cohort were graphically divided into three groups: oropharynx-like, skin-like, and atypical, based on their spatial proximity to the clusters mentioned above.

**Fig. 4:**
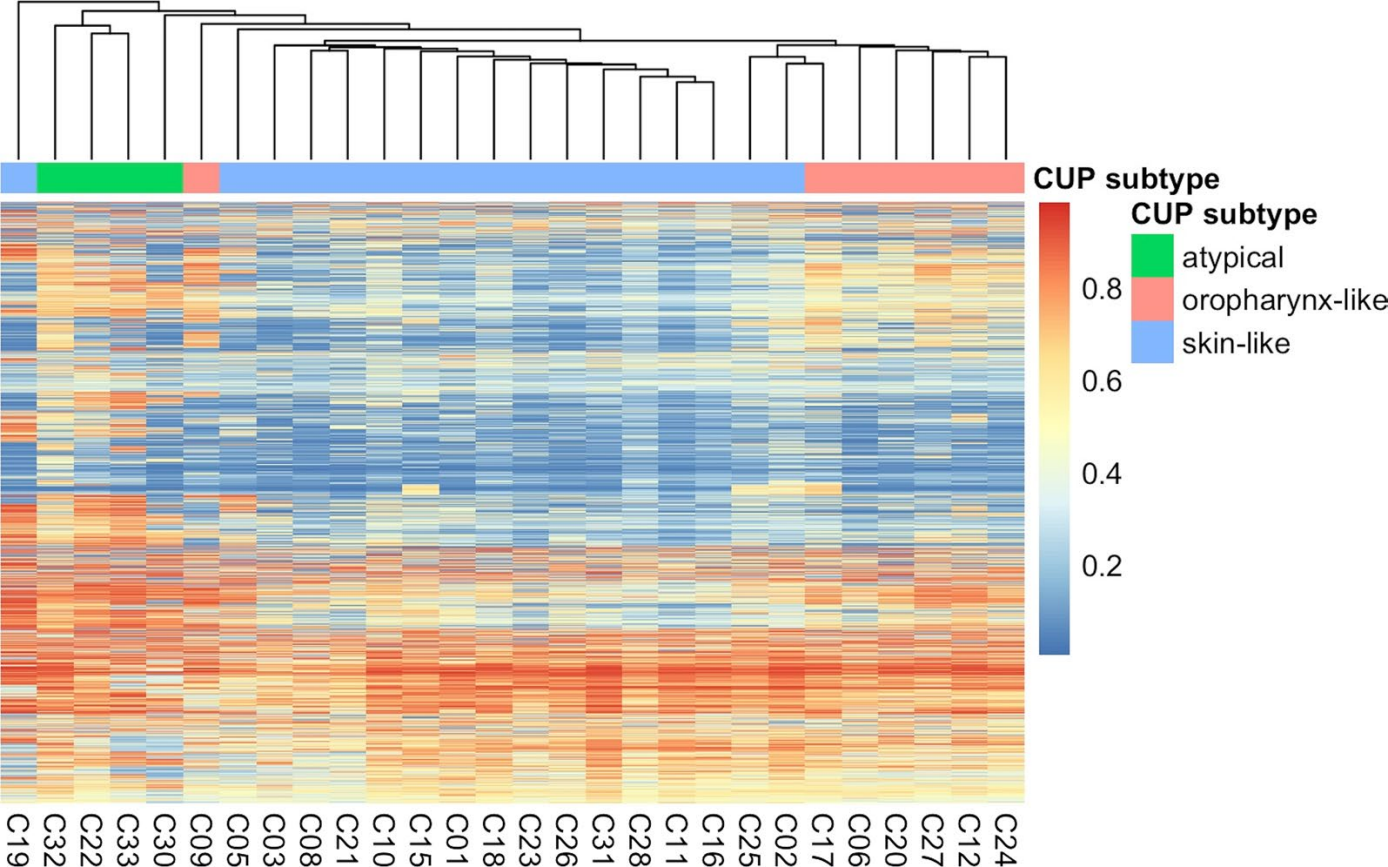
Hierarchical clustering of the hnCUP samples’ DNA methylation profiles. CUP subtypes and corresponding colors are matching those in Fig. 2. The 3,000 CpG sites (see Additional file: 3) are displayed per sample with their color-coded beta values

The hierarchical clustering of the hnCUP samples’ DNA methylation profiles was visualized with a dendrogram and heatmap in Fig. 4. The similarity of most oropharynx-like and skin-like samples can be clearly seen. Some of the skin-like samples’ methylation profiles show a hypomethylation across the 3000 selected CpG sites indicated by the blue color.

There was a significant difference between skin-like, oropharynx-like, and atypical samples in the distribution of the HPV status (Fisher’s exact test, *p* < 0.01) and nicotine consumption (Fisher’s exact test, *p* < 0.02). In contrast, no significant differences regarding other clinical variables like age and sex could be detected. Additional files 5 and 9 show the results in a table and the R script used to generate them, respectively.

### A classifier can predict tissue of origin based on DNA methylation with reasonable accuracy

**Fig. 5:**
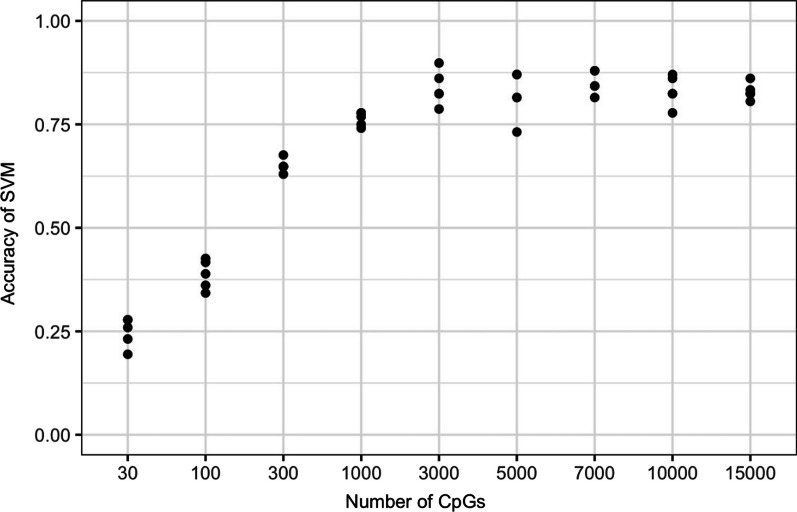
Accuracy of the SVM classifier at different numbers of CpG sites used for training

We used the training cohort to implement a classifier that can predict the tumor primary site of the test cohort by using its DNA methylation profiles. We compared an SVM with a random forest classifier. The SVM achieved a higher accuracy of up to 87%, compared to up to 75% for the random forest classifier, depending on the node size used (Additional file: 4). The SVM classifier was implemented using different amounts of CpG sites (10 to 15,000), yielding different overall accuracies (Fig. 5). For the final classifier, we used 3,000 CpG sites, as a larger set did not amount to a higher accuracy of the SVM as graphically depicted in Fig. 5.

The balanced accuracy defined as (sensitivity + specificity) / 2 was calculated to assess the performance of single-class prediction. As depicted in the confusion matrix (Table 5), the SVM classifier has a high balanced accuracy for the primary tumor sites: lung, skin, and lymphoma (92–100%). The entities forming the large cluster in Fig. 2 were predicted with a lower accuracy (86–90%). All lymphoma samples were classified correctly, and no non-lymphoma samples were misclassified as lymphoma. This demonstrates that methylation-based classification can be highly accurate even when applied to tissues of different origins, such as lymphatic versus squamous cell tissue.

**Table 5:** Confusion matrix for predicting the SVM classifier for the test set (*n* = 383)

Prediction	True entity									
	Esophagus	Larynx	Lung	Lymphoma	Oral cavity	Oropharynx	Skin	Balanced Accuracy	Sensitivity	Specificity
Esophagus	**24**	1	1	0	1	1	0	0.87	0.75	0.99
Larynx	3	**31**	2	0	5	2	1	0.86	0.76	0.96
Lung	1	3	**132**	0	3	0	0	0.97	0.98	0.97
Lymphoma	0	0	0	**16**	0	0	0	1.00	1.00	1.00
Oral cavity	3	5	0	0	**97**	5	0	0.90	0.85	0.95
Oropharynx	1	1	0	0	7	**25**	1	0.86	0.76	0.97
Skin	0	0	0	0	1	0	**10**	0.92	0.83	1.00

The true entity of the cohorts’ samples is displayed, and correct predictions are marked in bold. The balanced accuracy is shown as a performance parameter in single-class prediction

### The classifier predicts the primary site of hnCUP samples

The SVM classifier predicts the primary site of hnCUP samples used, as displayed in Table 6. Compared with the expected distribution (Table 4), no significant difference could be detected (*p* = 0.2, Pearson’s Chi-squared test). Samples whose primary site was predicted as oropharynx and skin all match the graphically determined oropharynx-like and skin-like samples (Fig. 3).

**Table 6:** Prediction of hnCUP samples by SVM classifier

Primary tumor site	Number of samples
Esophagus	1
Larynx	7
Lung	0
Lymphoma	0
Oral Cavity	11
Oropharynx	7
Skin	2
Overall	28

## Discussion

This study showed that DNA methylation profiles of hnCUP tissue are comparable to those of various SCCs known to metastasize to cervical lymph nodes. Based on tissue DNA methylation, we developed an SVM classifier that can distinguish these most commonly cervically metastasizing cancers. It is reasonably accurate (87%) and applicable to hnCUP samples. The distribution of predicted primary sites of hnCUP samples did not differ from the expected values based on published epidemiological data. Prior to our work, very few studies have been published on methylation data in hnCUP [14]—the examination of the samples in this study is a novelty in this field.

Despite state-of-the-art extensive diagnostic algorithms in the hospital, not in every hnCUP patient the underlying cancer entity can be specified after a thorough diagnostic workup [33].

It cannot be entirely ruled out that hnCUP is an entity of its own. However, our own data as well as other studies [2, 10, 13] strongly suggest that hnCUP is basically not a separate cancer entity but rather a metastatic disease. Our finding that the methylation levels of hnCUP samples are comparable to those of different SCCs that metastasize to cervical lymph nodes underscores the relevance of such methylation data.

The high specificity of the single-class predictors (see Table 5) is clinically relevant. The low rate of falsely predicted tumor sites would make invasive diagnostics and therapy at the wrong anatomical site unlikely. The specificity of over 90% achieved across all examined primary tumor sites would suffice to use a classifier reliably in a clinical setting.

Our research indicates that DNA methylation profiles of CSCC showed similarities to our hnCUP samples **(**Fig. 3**)**. Therefore, CSCC might be an overlooked entity when searching for primary sites of hnCUP. Despite routine skin examinations in the diagnostic process, small primary tumors or ones affecting the haired scalp are easily left unnoticed [34].

On the other hand, the classifier predicted only one hnCUP sample to be originating from the esophagus. Based on our analysis of the occurrence of cervical lymph node metastasis, we would have statistically expected to locate six primaries in the esophagus. Possibly, even small esophageal carcinomas are more easily diagnosed in the smoothly surfaced mucosa with modern techniques [35] compared to, e.g., the poorly visible crypts in the tonsils or tongue base [36]. Lymph node metastasis in ESCC is associated with advanced-stage tumors [37], which could also contribute to this observation.

To calculate the expected proportion of origins for head and neck SCC lymph node metastasis, the worldwide incidence rates and rates of cervical metastasis were used since no data on the proportion are available [21–26]. However, it must be noted that metastasis with loss of the primary tumor site, as is presumed to be the case in a proportion of hnCUP cases, may be subject to different epidemiology. In addition, the calculated incidence rates in Table 4 are only a rough estimate. They would be more accurately described based on the anatomic localization of the metastasis since lymph nodes from ESCC are more commonly found in neck level 4 [38]. In contrast, oropharyngeal carcinoma metastasizes in early stages, primarily in neck levels 2 and 3 [39].

The hnCUP, CSCC, and HNSCC samples from Leitheiser et al. [14] were analyzed using the Illumina EPIC chip. While appropriate methods have been used for cross-normalization with probes from the Illumina 450 k chip [31], the similarity between these entities on methylation level could partly be explained by the different chip used. In contrast, other studies have suggested that the impact is likely negligible when the analysis does not solely focus on individual methylation sites [40, 41].

Another limitation might be non-tumor cells interfering with the methylation data. Our plots and classifier reveal notable differences in methylation between entities within the selected CpG sites, as has been demonstrated in other studies as well [14, 17, 19]. Achieving significantly higher percentages of tumor proportion may require the application of single-cell methods [42].

The classifier predicted the localization of the primary with 87% accuracy. With the current best diagnostic method, FDG-PET-CT, only 29% of additional primaries were found after extensive diagnostic workup with panendoscopy and CT/MRI scans in a prospective study [43], underlining the relevance of our algorithm. In patients with a p16/HPV-positive hnCUP, the literature shows a strong association with a primary in the oropharynx and a better clinical outcome [44, 45]. The clinically challenging cases are p16/HPV-negative cases with a poorer prognosis. Therefore, narrowing down the localization would be especially relevant in these HPV-negative cases. Using a classifier could assist in finding the primary and avoid unnecessary procedures in cases with a suspected primary in the skin or esophagus. However, the classifier was based on methylation data from primary tumors, not lymph node metastasis. A further validation study examining primary tumor and lymph node metastasis tissue would be needed, which could even increase the accuracy of the classifier. The classifier could also be tested in a prospective study in cases with initial suspicion of hnCUP.

In the context of training a machine learning-based classifier, using a limited sample size in the training set can potentially lead to challenges in achieving accurate classification [46]. Only *n* = 25, CSCC samples were available for training. However, higher case numbers could seriously improve our SVM classifier’s capacity to decipher the epigenetic variations within their DNA methylation profiles. The differences between CSCC samples at the level of DNA methylation might demand a more expansive data set to enable the SVM model to fully capture the breadth of this entity. This scarcity in sample size underscores the critical need for a more extensive training data set containing more DNA methylation profiles of CSCC. This would be very valuable for enhancing the precision and robustness of the classification model in accurately categorizing hnCUP samples based on their DNA methylation profiles.

Some skin-like hnCUP samples showed hypomethylation across the 3,000 CpG sites used for our analyses (Fig. 4). Global hypomethylation is a phenomenon commonly observed in human tumors and, while generally poorly understood, appears to occur parallel to the de novo methylation of tumor suppressors, a known driver of tumorigenesis [47]. This hypomethylation could help to stratify hnCUP into subgroups and might be another pillar in differentiating squamous cell carcinomas of various origins.

## Conclusion

Tumor tissue samples of hnCUP patients are comparable to other cancer entities that commonly metastasize to cervical lymph nodes on DNA methylation level. An SVM-based classifier can accurately distinguish these cancers. Our approach could significantly reduce the invasiveness and side effects of diagnostic and therapeutic procedures in hnCUP. A prospective study is the next step in translating our classifier into clinical practice.

### Supplementary Information

**Additional file 1 **This file contains five Additional file figures showing a UMAP plot of the two-third training split with the actual tumor entity (slide 1) and a UMAP plot of the one-third test split with the tumor site predicted by the support vector machine (slide 2). **Figs**. S3 and S4 (slides 3 and 4) show alternative versions of **Fig. S2 **using PCA and t-SNE for dimensionality reduction instead of UMAP. The fifth slide includes a plot visualizing the batch effect of the methylation array used and the centers where the analyses were performed.

**Additional file 2** This file contains clinical data of the study cohort.

**Additional file 3 ** This file contains the names of the 3000 CpG sites used for classifier development.

**Additional file 4** The accuracies of the random forest classifiers using different node sizes (1, 2, 3, 10, 50) are included in this file.

**Additional file 5 **This file contains the statistical tests and the p-values used to compare the methylation subgroups with various clinical variables.

**Additional file 6** This file contains the R script used for data preprocessing and normalization.

**Additional file 7 ** Figures shown in the manuscript (R Script).

**Additional file 8** The implementation of the classifier used (R Script).

**Additional file 9 **This R script generated the results shown in Additional file: 5.

## Data Availability

The microarray methylation data from this study have been deposited in the NCBI Gene Expression Omnibus under accession number GSE256413.

## Abbreviations

CUP: Cancer of unknown primary
CSCC: Cutaneous squamous cell carcinoma
DCBL: Diffuse large B-cell lymphoma
DKFZ: Deutsches Krebsforschungszentrum (German cancer research center)
ESCC: Esophageal squamous cell carcinoma
FFPE: Formalin-fixed and paraffin-embedded
hnCUP: Head and neck cancer of unknown primary
HNSCC: Head and neck squamous cell carcinoma
HPV: Human papillomavirus
LUSCC: Lung squamous cell carcinoma
NIH: National Institutes of Health
OCSCC: Oral cavity squamous cell carcinoma
PCA: Principal component analysis
SCC: Squamous cell carcinoma
SVM: Support vector machine
TCGA: The cancer genome atlas
*t*-SNE: *t*-Distributed stochastic neighbor embedding
UMAP: Uniform manifold approximation and projection

## References

[1] Sung H, Ferlay J, Siegel RL, Laversanne M, Soerjomataram I, Jemal A (2021). Global cancer statistics 2020: GLOBOCAN estimates of incidence and mortality worldwide for 36 cancers in 185 countries. CA Cancer J Clin.

[2] Grau C, Johansen LV, Jakobsen J, Geertsen P, Andersen E, Jensen BB (2000). Cervical lymph node metastases from unknown primary tumours: results from a national survey by the Danish society for head and neck oncology. Radiother Oncol.

[3] Cianchetti M, Mancuso AA, Amdur RJ, Werning JW, Kirwan J, Morris CG (2009). Diagnostic evaluation of squamous cell carcinoma metastatic to cervical lymph nodes from an unknown head and neck primary site. Laryngoscope.

[4] Strojan P, Ferlito A, Medina JE, Woolgar JA, Rinaldo A, Robbins KT (2013). Contemporary management of lymph node metastases from an unknown primary to the neck: I. A review of diagnostic approaches. Head Neck.

[5] Strojan P, Anicin A (1998). Combined surgery and postoperative radiotherapy for cervical lymph node metastases from an unknown primary tumour. Radiother Oncol.

[6] Regelink G, Brouwer J, de Bree R, Pruim J, van der Laan BF, Vaalburg W (2002). Detection of unknown primary tumours and distant metastases in patients with cervical metastases: value of FDG-PET versus conventional modalities. Eur J Nucl Med Mol Imaging.

[7] Issing WJ, Taleban B, Tauber S (2003). Diagnosis and management of carcinoma of unknown primary in the head and neck. Eur Arch Otorhinolaryngol.

[8] Conway AM, Mitchell C, Kilgour E, Brady G, Dive C, Cook N (2019). Molecular characterisation and liquid biomarkers in Carcinoma of unknown primary (CUP): taking the 'U' out of 'CUP'. Br J Cancer.

[9] Gottschlich S, Schuhmacher O, Görögh T, Hoffmann M, Maune S (2000). Analysis of the p53 gene status of lymph node metastasis in the head and neck region in occult primary cancer. Laryngorhinootologie.

[10] Pavlidis N, Pentheroudakis G (2012). Cancer of unknown primary site. The Lancet.

[11] Möhrmann L, Werner M, Oleś M, Mock A, Uhrig S, Jahn A (2022). Comprehensive genomic and epigenomic analysis in cancer of unknown primary guides molecularly-informed therapies despite heterogeneity. Nat Commun.

[12] Greco FA, Erlander MG (2009). Molecular classification of cancers of unknown primary site. Mol Diagn Ther.

[13] Alshareeda AT, Al-Sowayan BS, Alkharji RR, Aldosari SM, Al Subayyil AM, Alghuwainem A (2020). Cancer of unknown primary site: real entity or misdiagnosed disease?. J Cancer.

[14] Leitheiser M, Capper D, Seegerer P, Lehmann A, Schüller U, Müller KR (2022). Machine learning models predict the primary sites of head and neck squamous cell carcinoma metastases based on DNA methylation. J Pathol.

[15] Lorkowski SW, Dermawan JK, Rubin BP (2024). The practical utility of AI-assisted molecular profiling in the diagnosis and management of cancer of unknown primary: an updated review. Virchows Arch.

[16] Koelsche C, Schrimpf D, Stichel D, Sill M, Sahm F, Reuss DE (2021). Sarcoma classification by DNA methylation profiling. Nat Commun.

[17] Sahm F, Schrimpf D, Stichel D, Jones DTW, Hielscher T, Schefzyk S (2017). DNA methylation-based classification and grading system for meningioma: a multicentre, retrospective analysis. Lancet Oncol.

[18] Roohani S, Ehret F, Perez E, Capper D, Jarosch A, Flörcken A (2022). Sarcoma classification by DNA methylation profiling in clinical everyday life: the Charité experience. Clin Epigenet.

[19] Moran S, Martinez-Cardus A, Sayols S, Musulen E, Balana C, Estival-Gonzalez A (2016). Epigenetic profiling to classify cancer of unknown primary: a multicentre, retrospective analysis. Lancet Oncol.

[20] Jurmeister P, Bockmayr M, Seegerer P, Bockmayr T, Treue D, Montavon G (2019). Machine learning analysis of DNA methylation profiles distinguishes primary lung squamous cell carcinomas from head and neck metastases. Sci Transl Med.

[21] López F, Rodrigo JP, Silver CE, Haigentz M, Bishop JA, Strojan P (2016). Cervical lymph node metastases from remote primary tumor sites. Head Neck.

[22] Ferlay J, Shin H, Bray F, Forman D, Mathers C, Parkin D (2010). Estimates of worldwide burden of cancer in 2008: GLOBOCAN 200. Int J Cancer.

[23] Nakagawa S, Nishimaki T, Kosugi S, Ohashi M, Kanda T, Hatakeyama K (2003). Cervical lymphadenectomy is beneficial for patients with carcinoma of the upper and mid-thoracic esophagus. Dis Esophagus.

[24] Wenzel S, Sagowski C, Kehrl W, Metternich F (2004). The prognostic impact of metastatic pattern of lymph nodes in patients with oral and oropharyngeal squamous cell carcinomas. Eur Arch Oto-Rhino-Laryngol Head Neck.

[25] Zhu X, Zhao M, Zhou L, Zhang M, Cao P, Tao L (2020). Significance of examined lymph nodes number and metastatic lymph nodes ratio in overall survival and adjuvant treatment decision in resected laryngeal carcinoma. Cancer Med.

[26] Karia PS, Han J, Schmults CD (2013). Cutaneous squamous cell carcinoma: estimated incidence of disease, nodal metastasis, and deaths from disease in the United States, 2012. J Am Acad Dermatol.

[27] NIH. The cancer genome atlas program—national cancer institute. 2020.

[28] Rodríguez-Paredes M, Bormann F, Raddatz G, Gutekunst J, Lucena-Porcel C, Köhler F (2018). Methylation profiling identifies two subclasses of squamous cell carcinoma related to distinct cells of origin. Nat Commun.

[29] Koelsche C, Stichel D, Griewank KG, Schrimpf D, Reuss DE, Bewerunge-Hudler M (2019). Genome–wide methylation profiling and copy number analysis in atypical fibroxanthomas and pleomorphic dermal sarcomas indicate a similar molecular phenotype. Clin Sarcoma Res.

[30] Aryee MJ, Jaffe AE, Corrada-Bravo H, Ladd-Acosta C, Feinberg AP, Hansen KD (2014). Minfi: a flexible and comprehensive bioconductor package for the analysis of Infinium DNA methylation microarrays. Bioinformatics.

[31] Fortin JP, Triche TJ, Hansen KD (2017). Preprocessing, normalization and integration of the illumina humanmethylationEPIC array with minfi. Bioinformatics.

[32] Didona D, Paolino G, Bottoni U, Cantisani C (2018). Non melanoma skin cancer pathogenesis overview. Biomedicines.

[33] Pinkiewicz M, Dorobisz K, Zatoński T (2021). A systematic review of cancer of unknown primary in the head and neck region. Cancer Manag Res.

[34] Kawaguchi M, Kato H, Matsuo M (2019). CT and MRI features of scalp lesions. Radiol Med.

[35] Yip HC, Chiu PW (2017). Endoscopic diagnosis and management of early squamous cell carcinoma of esophagus. J Thorac Dis.

[36] Buckley L, Gupta R, Ashford B, Jabbour J, Clark JR (2016). Oropharyngeal cancer and human papilloma virus: evolving diagnostic and management paradigms. ANZ J Surg.

[37] Wang H, Deng F, Liu Q, Ma Y (2017). Prognostic significance of lymph node metastasis in esophageal squamous cell carcinoma. Pathol Res Pract.

[38] Kang Y, Hwang Y, Lee HJ, Park IK, Kim YT, Kang CH (2017). Patterns and prognostic significance of cervical lymph node metastasis and the efficacy of cervical node dissection in esophageal cancer. Korean J Thorac Cardiovasc Surg.

[39] Lindberg R (1972). Distribution of cervical lymph node metastases from squamous cell carcinoma of the upper respiratory and digestive tracts. Cancer.

[40] Cheung K, Burgers MJ, Young DA, Cockell S, Reynard LN (2020). Correlation of infinium humanmethylation450K and MethylationEPIC BeadChip arrays in cartilage. Epigenetics.

[41] Fernandez-Jimenez N, Allard C, Bouchard L, Perron P, Bustamante M, Bilbao JR (2019). Comparison of Illumina 450K and EPIC arrays in placental DNA methylation. Epigenetics.

[42] Smallwood SA, Lee HJ, Angermueller C, Krueger F, Saadeh H, Peat J (2014). Single-cell genome–wide bisulfite sequencing for assessing epigenetic heterogeneity. Nat Methods.

[43] Johansen J, Buus S, Loft A, Keiding S, Overgaard M, Hansen HS (2008). Prospective study of 18FDG-PET in the detection and management of patients with lymph node metastases to the neck from an unknown primary tumor. Results from the DAHANCA-13 study. Head Neck.

[44] Fotopoulos G, Pavlidis N (2015). The role of human papilloma virus and p16 in occult primary of the head and neck: a comprehensive review of the literature. Oral Oncol.

[45] Leemans CR, Snijders PJF, Brakenhoff RH (2018). The molecular landscape of head and neck cancer. Nat Rev Cancer.

[46] Popovici V, Chen W, Gallas BD, Hatzis C, Shi W, Samuelson FW (2010). Effect of training-sample size and classification difficulty on the accuracy of genomic predictors. Breast Cancer Res.

[47] Ehrlich M (2009). DNA hypomethylation in cancer cells. Epigenomics.

