# Supplementary material for: Head and neck cancer of unknown primary: unveiling primary tumor sites through machine learning on DNA methylation profiles
Source: Clin Epigenetics. 2024 Mar 25;16:47. doi: 10.1186/s13148-024-01657-3 (PMC10964705; doi:10.1186/s13148-024-01657-3)
Supplement: Supplementary file 1 — Additional file 1 This file contains five Additional file figures showing a UMAP plot of the two-third training split with the actual tumor entity (slide 1) and a UMAP plot of the one-third test split with the tumor site predicted by the support vector machine (slide 2). Figs. S3 and S4 (slides 3 and 4) show alternative versions of Fig. S2 using PCA and t-SNE for dimensionality reduction instead of UMAP. The fifth slide includes a plot visualizing the batch effect of the methylation array used and the centers where the analyses were performed. [file 13148_2024_1657_MOESM1_ESM.pptx]

## Slide 1
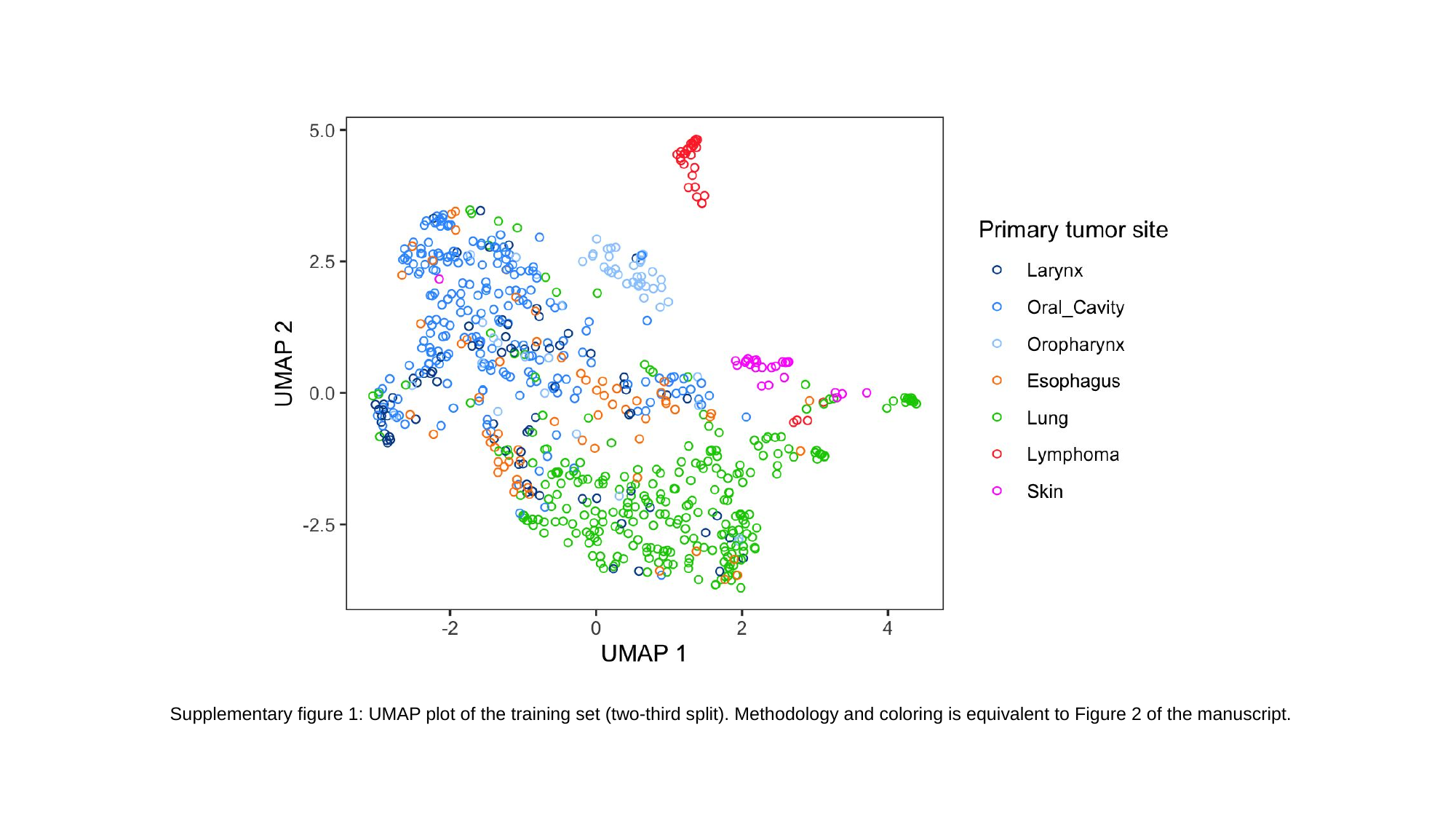

Supplementary figure 1: UMAP plot of the training set (two-third split). Methodology and coloring is equivalent to Figure 2 of the manuscript.

## Slide 2
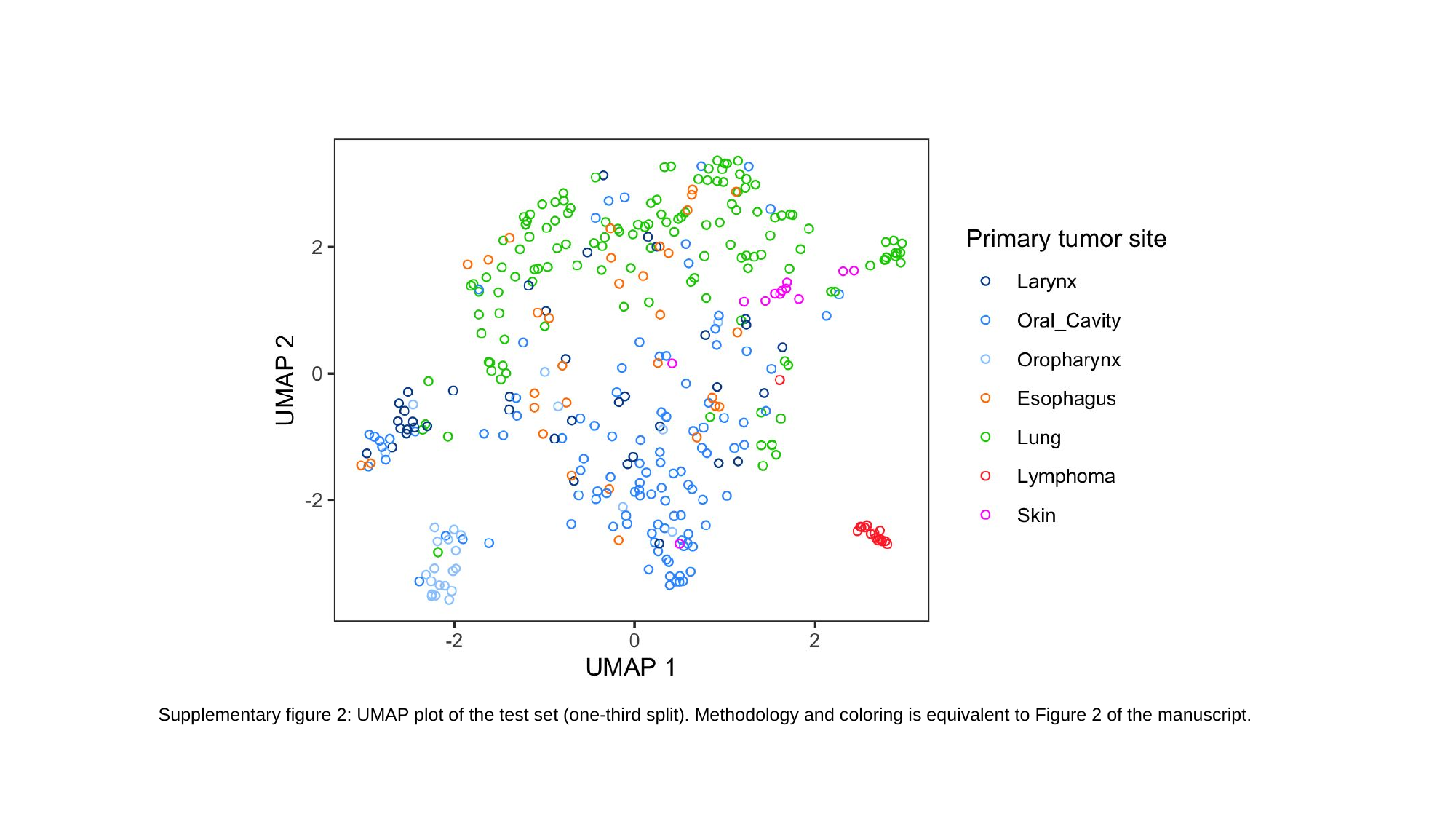

Supplementary figure 2: UMAP plot of the test set (one-third split). Methodology and coloring is equivalent to Figure 2 of the manuscript.

## Slide 3
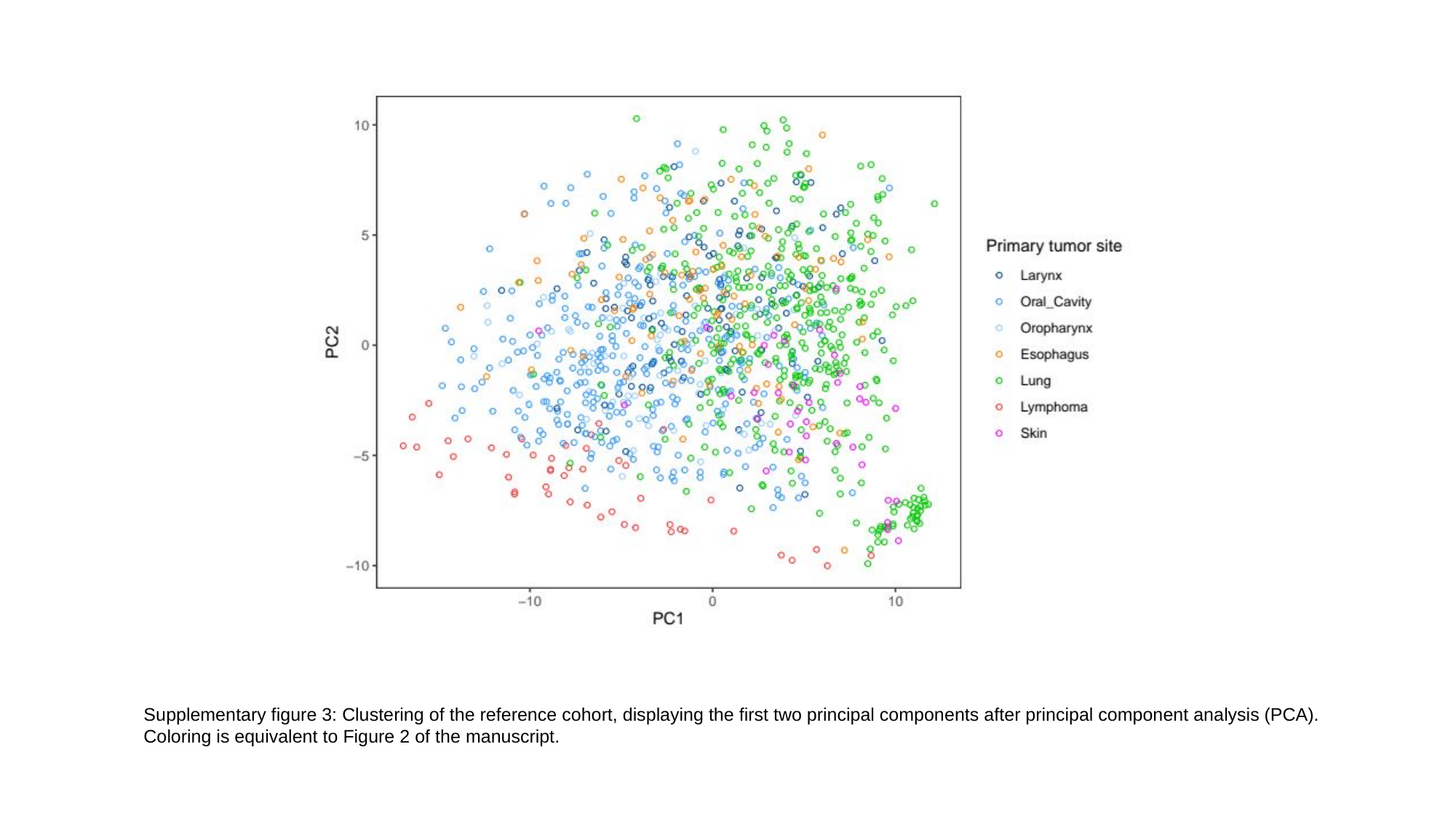

Supplementary figure 3: Clustering of the reference cohort, displaying the first two principal components after principal component analysis (PCA).
Coloring is equivalent to Figure 2 of the manuscript.

## Slide 4
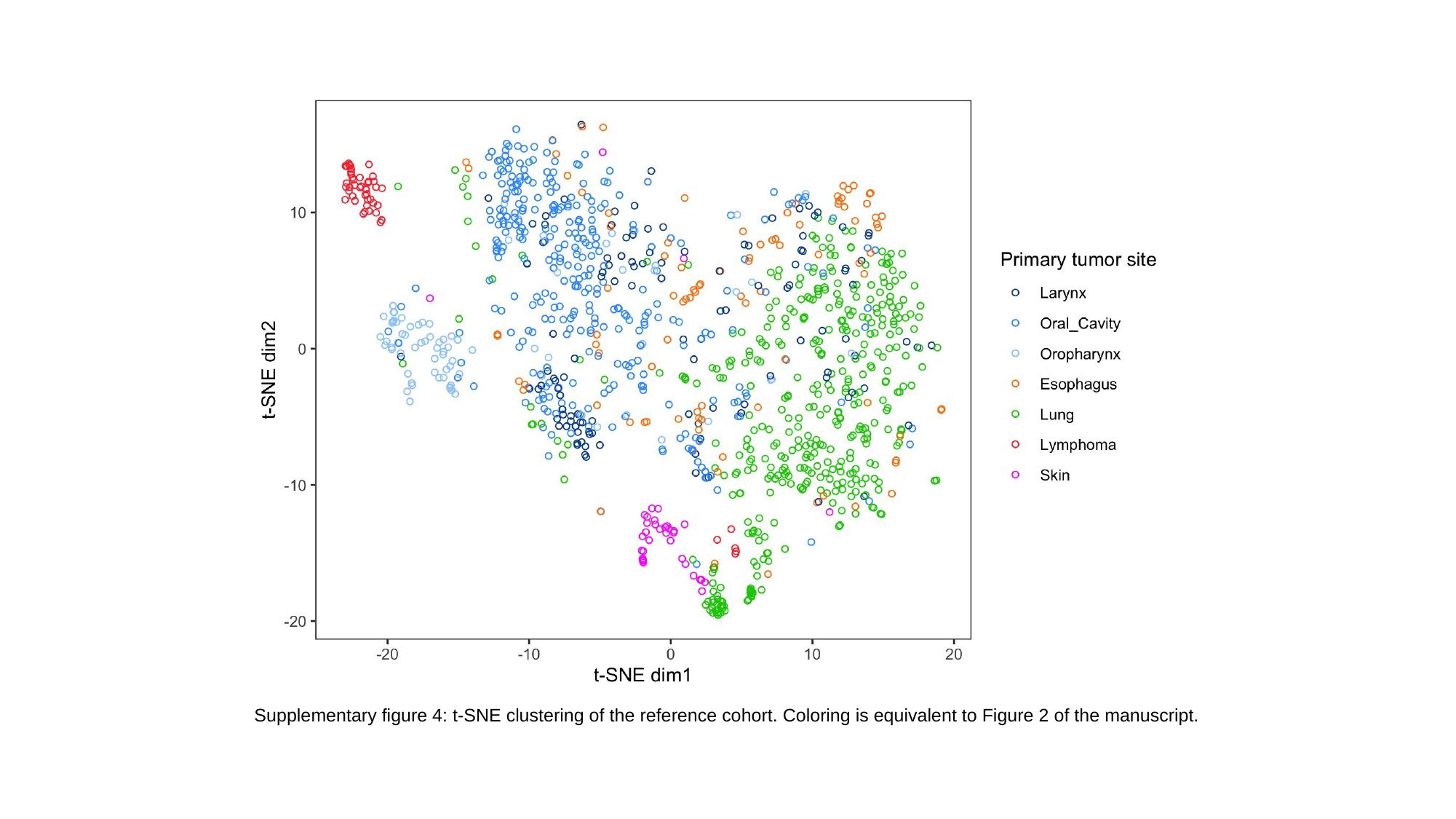

Supplementary figure 4: t-SNE clustering of the reference cohort. Coloring is equivalent to Figure 2 of the manuscript.

## Slide 5
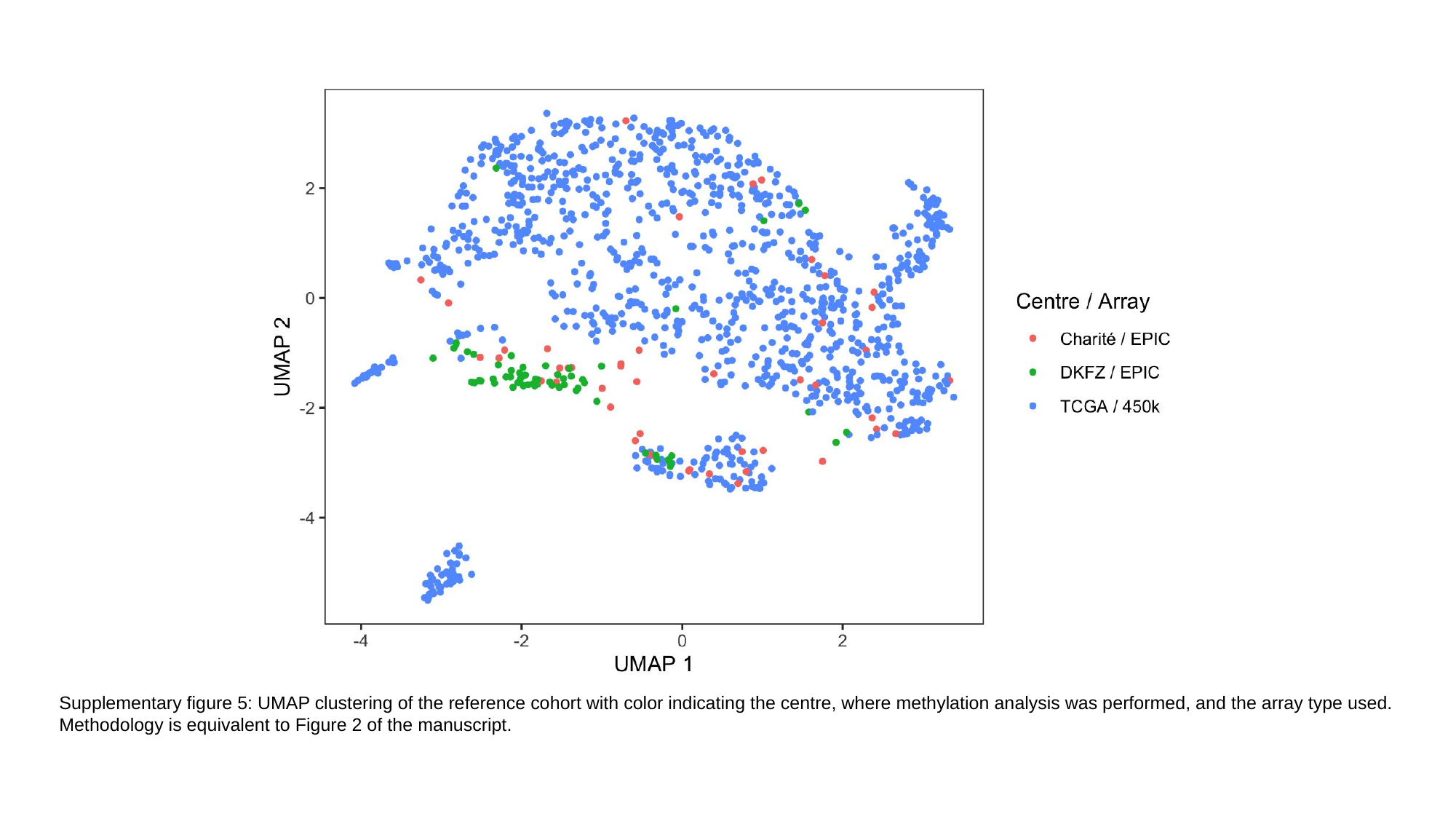

Supplementary figure 5: UMAP clustering of the reference cohort with color indicating the centre, where methylation analysis was performed, and the array type used.
Methodology is equivalent to Figure 2 of the manuscript.
